# SARS-CoV-2 screening testing in schools for children with intellectual and developmental disabilities

**DOI:** 10.1186/s11689-021-09376-z

**Published:** 2021-09-01

**Authors:** Michael R. Sherby, Tyler J. Walsh, Albert M. Lai, Julie A. Neidich, Joyce E. Balls-Berry, Stephanie M. Morris, Richard Head, Christopher G. Prener, Jason G. Newland, Christina A. Gurnett, for the COMPASS-T Study Group, Megan Baldenweck, Kelly Bono, Victor B. Brodsky, Charlene A. Caburnay, John N. Constantino, Nikole Lobb Dougherty, James M. Dubois, Stephanie A. Fritz, George S. Gotto, Adwoa Imbeah, Luther G. Kalb, Jingxia Liu, Brett B. Maricque, Virginia R. McKay, Linda S. Myers, Timothy J. Poor, Byron J. Powell, Nancy B. Mueller, Bradley L. Schlaggar, Ann Schmidt, Elsa Snider, Matthew C. Traughber, Maureen van Stone, Liz Vestal, Myisha Wilcher-Roberts

**Affiliations:** 1 Department of Neurology, Division of Pediatric and Developmental Neurology, Washington University in St. Louis, 660 S. Euclid Avenue Campus, Box 8111, St. Louis, MO 63110 USA; 2 Department of Pediatrics, Division of Pediatric Infectious Diseases, Washington University in St. Louis, St. Louis, MO USA; 3 Department of Medicine, Washington University in St. Louis, St. Louis, MO USA; 4 Department of Pathology and Immunology, Washington University in St. Louis, St. Louis, MO USA; 5 Department of Neurology, Division of Memory and Aging, Washington University in St. Louis, St. Louis, MO USA; 6 Department of Genetics, Washington University in St. Louis, St. Louis, MO USA; 7 Department of Sociology and Anthropology, Saint Louis University, St. Louis, MO USA

**Keywords:** SARS-CoV-2 testing, COVID-19, COVID-19 School tests, Children with IDD, Intellectual and developmental disabilities

## Abstract

**Background:**

Transmission of SARS-CoV-2 in schools primarily for typically developing children is rare. However, less is known about transmission in schools for children with intellectual and developmental disabilities (IDD), who are often unable to mask or maintain social distancing. The objectives of this study were to determine SARS-CoV-2 positivity and in-school transmission rates using weekly screening tests for school staff and students and describe the concurrent deployment of mitigation strategies in six schools for children with IDD.

**Methods:**

From November 23, 2020, to May, 28, 2021, weekly voluntary screening for SARS-CoV-2 with a high sensitivity molecular-based saliva test was offered to school staff and students. Weekly positivity rates were determined and compared to local healthcare system and undergraduate student screening data. School-based transmission was assessed among participants quarantined for in-school exposure. School administrators completed a standardized survey to assess school mitigation strategies.

**Results:**

A total of 59 students and 416 staff participated. An average of 304 school staff and students were tested per week. Of 7289 tests performed, 21 (0.29%) new SARS-CoV-2 positive cases were identified. The highest weekly positivity rate was 1.2% (*n* = 4) across all schools, which was less than community positivity rates. Two cases of in-school transmission were identified, each among staff, representing 2% (2/103) of participants quarantined for in-school exposure. Mitigation strategies included higher than expected student mask compliance, reduced room capacity, and phased reopening.

**Conclusions:**

During 24 weeks that included the peak of the COVID-19 pandemic in winter 2020-21, we found lower rates of SARS-CoV-2 screening test positivity among staff and students of six schools for children with IDD compared to community rates. In-school transmission of SARS-CoV-2 was low among those quarantined for in-school exposure. However, the impact of the emerging SARS-CoV-2 Delta variant on the effectiveness of these proven mitigation strategies remains unknown.

**Trial registration:**

Prior to enrollment, this study was registered at ClinicalTrials.gov on September 25, 2020, identifier NCT04565509, titled Supporting the Health and Well-being of Children with Intellectual and Developmental Disability During COVID-19 Pandemic.

**Supplementary Information:**

The online version contains supplementary material available at 10.1186/s11689-021-09376-z.

## Background

In-person school for children with intellectual and developmental disabilities (IDD) provides daily structure, health care services, and therapy, in addition to education. Approximately 14% of all public-school students have disabilities, which includes students with learning disabilities, speech and language impairment, autism, intellectual disabilities, and emotional disturbance [1]. The American Academy of Pediatrics advocates that in-person learning is particularly important for these children with disabilities due to the additional benefits in-person school provides [2]. During the COVID-19 pandemic, parents/guardians of children with IDD have anecdotally reported being overburdened by taking on additional roles (e.g., physical therapy) that school previously provided. Even pre-pandemic, parents/guardians of children with IDD reported more parenting stress than parents/guardians of typically developing children [3, 4]. Stress can have a compound effect, with challenging child behaviors impacting parental adjustment, which in turn impacts the child’s behavior [5]. However, because children with IDD are more vulnerable to severe acute respiratory syndrome coronavirus-2 (SARS-CoV-2) infection [6], with fatality rates reported as high as 1.6% [7], many parents/guardians have been reluctant for them to resume in-person learning.

Despite the accepted benefits of in-person school, teaching children with IDD presents challenges that could increase the risk of SARS-CoV-2 infection to school staff (teachers, administrators, and staff) and students. Appropriate mask wearing, a foundational school mitigation strategy [8, 9], may be challenging for many children with IDD. Many disabled students require assistance with activities of daily living, such as eating, during which social distancing and masking cannot occur. While general recommendations are provided for safe return to school [10, 11], specific guidance is unavailable for schools dedicated to children with IDD. Furthermore, since many of these schools remained virtual throughout the pandemic, mitigation strategies specifically targeted to these schools have not yet been evaluated.

SARS-CoV-2 screening is a mitigation strategy recommended by the Centers for Disease Control and Prevention (CDC) when community transmission is moderate, substantial, or high, based on specific thresholds [10]. Literature suggests that screening testing in a typical school setting is unlikely to provide additional benefit even when community transmission is high, based on low rates of school-based SARS-CoV-2 transmission [8, 9, 12]. However, the utility of screening tests in schools for children with IDD, which may be considered high-risk environments for transmission, has not yet been determined.

The objectives of this study were to evaluate the impact of SARS-CoV-2 weekly screening for school staff and students on in-school transmission and describe the concurrent deployment of mitigation strategies in six schools for children with IDD.

## Methods

### Study population

The study took place at six schools dedicated to children with IDD within the Special School District (SSD) of St. Louis County. SSD provides special education and related services for more than 23,000 students within 22 school districts in St. Louis County. While the vast majority of these students attend school in the district in which they live, 716 children with IDD ages 5–21 years are educated in one of the district’s six special education schools. SSD students’ medical needs are complex, including 54 students with non-progressive neuromuscular disorder, 8 with progressive neuromuscular disorder, 90 with permanent orthopedic disabilities, 42 receiving gastric-tube feedings, and 11 with tracheostomies. Children who attend these schools are generally bussed from their homes daily. These schools employ 605 teachers, staff, and administrators.

All staff and students were invited to participate in the study, during which weekly saliva samples were collected for SARS-CoV-2 screening testing. Staff, student participants 18 and over, and parent/guardian of students under 18 provided written consent and completed an intake survey of demographic and health data. Here, we report the results of weekly screening of testing starting November 23, 2020, through the end of the school year, May 28, 2021. Student testing did not start until December 11, 2020.

For community level data, we collected ZIP code data from St. Louis City and County public dashboards during the pandemic beginning in early April 2020. Counts were associated with estimated populations for ZIP Code Tabulation Areas (ZCTA) to create new case infection rates for 3-month periods. Since the number of cases identified during these periods varies widely, data were converted to quintiles to facilitate comparisons between periods [13].

### In-school screening testing

A highly sensitive PCR-based assay to detect SARS-CoV-2 in saliva was developed by Washington University investigators at the McDonnell Genome Institute in partnership with Fluidigm [14]. Each SSD school was assigned a day of the week throughout the study during which samples were collected from participants. Staff predominantly collected their samples at home and submitted them to the study team at a predefined location in the school building prior to the start of the school day. Student collection occurred at the beginning of the school day either in the nurse’s office, classroom, or predefined testing location within the school. The study coordinators, with assistance from nurses and teachers, collected the samples from the students. Participants were instructed to submit the test each week even if they had been quarantined, had symptoms, or had tested positive the previous week. For these situations, the tests were collected in the school parking lot. The assay was performed at Washington University’s Genome Technology Access Center Cortex laboratory as a CAP/CLIA diagnostic test with clinical oversight by Pathology and Immunology. Results were returned to the participants and the study team on the same day samples were collected. Negative test results were reported by an automated email. Positive results were reported directly to participants by phone from one of the study Principal Investigators (JN).

### Evaluation of positive cases

Staff and students testing positive for SARS-CoV-2 were immediately isolated for at least 10 days, in compliance with the St. Louis County Department of Public Health and SSD human resource policies. All positive participants or their parents/legal guardians were interviewed to determine potential exposures to persons with COVID-19 and to identify potential in-school contacts. Contact tracing was conducted by SSD, as this task was delegated to the schools in St. Louis County due to the high rates of community transmission.

### Outcome measures

The primary outcome was the weekly positivity rate in the six SSD schools. Additionally, we assessed the rate of school-based transmission among the participants who continued testing during their quarantine period. In-school SARS-CoV-2 transmission was determined by two trained investigators (JN and TW). Data utilized to make this determination included quarantine status, exposure date, diagnosis date, symptom onset, and potential alternative source of transmission (e.g., household contact). When a participant tested positive, the type of transmission was determined by identifying if the participant was currently being quarantined due to a school, household, or community exposure. Additionally, each positive participant underwent a standardized interview by JN to determine onset of symptoms, other potential exposures, and recent high-risk activities. After compiling these data, JN and TW determined if a positive participant transmission occurred in the school, household, community, or was unknown.

### School-level data

Administrators from SSD provided demographic data and completed a standardized survey on the mitigation strategies being implemented at each school. Additional positive SARS-CoV-2 cases among school staff and students not participating in the weekly screening testing were recorded.

### Analysis

We performed descriptive statistics to summarize participant data and school level data utilizing frequencies, percentages, medians, and interquartile ranges. Weekly positivity rates were calculated and aggregated. The school-based secondary transmission rate was calculated among participants.

## Results

The six SSD schools participating in this research study were located throughout St. Louis County (Supplemental Figure 1). The staff and students at the schools varied by race, in-person attendance, and percentage of students receiving free or reduced-price lunch (Table 1). During the first few months of the pandemic from March to June 2020 (see Supplemental Figure 1, inset), the highest rates of COVID-19 cases by quintile occurred in Midtown, North St. Louis City, and North St. Louis County. At study commencement in November 2020, the highest quintile of new cases was in South St. Louis County.

**Table 1:** Special School District of St. Louis County school demographics and mitigation strategies

	All Schools	School					
		1 (9-12+)	2 (K-8)	3 (9-12+)	4 (K-8)	5* (9-12+)	6* (K-8)
**Staff employed**	**622**	**109**	**105**	**120**	**142**	**70**	**76**
Teachers and aids	466	75	76	95	103	68	49
Administrative staff	2	4	4	4	4	5	5
Ancillary services	79	9	15	15	16	24	24
Nurses	20	2	7	2	6	3	3
Other	8	5	3	0	0	0	0
**Students enrolled** (week 14/week 24)	**723/656**	**118/95**	**132/128**	**163/132**	**172/173**	**79/73**	**59/55**
Black/African-American	378 (52)	42 (36)	69 (52)	119 (71)	136 (79)	5 (6)	7 (13)
White Non-Hispanic	295 (41)	72 (61)	44 (34)	40 (24)	32 (18)	67 (85)	40 (73)
Hispanic/Latino	20 (3)	3 (3)	3 (2)	5 (3)	3 (2)	3 (4)	3 (5)
Asian	17 (2)	1 (1)	10 (8)	0 (0)	0 (0)	3 (4)	3 (5)
Other	10 (1)	0 (0)	5 (4)	0 (0)	2 (1)	1 (1)	2 (4)
In-person/hybrid learning (week 1)	428 (59)	69 (59)	83 (63)	70 (42)	102 (58)	54 (68)	46 (82)
In-person learning (week 14)	467 (65)	76 (64)	93 (70)	97 (59)	98 (57)	61 (77)	49 (84)
In-person learning (week 24)	439 (67)	63 (66)	90 (70)	80 (61)	99 (57)	61 (84)	46 (84)
% receiving free or reduced lunch	–	33%	100%	100%	100%	27%	53%
**Mitigation strategies**							
% staff masked	–	100%	100%	100%	100%	> 75%	> 75%
% students masked	–	50–75%	> 75%	> 75%	> 75%	50–75%	50–75%
Desks spaced at least 6 feet in classrooms	–	No	Yes	No	Yes	No	No
Symptom screening for students	–	Yes	Yes	Yes	Yes	Yes	Yes
Barriers in place	–	Yes	Yes	No	Yes	Yes	Yes
Lunch location	–	Class	Class	Class	Class	Class	Class
Ventilation system replaced	–	No	No	No	No	No	No

Percentages are indicated in parenthesis

*Schools 5 and 6 are in the same building and share the same nurses, ancillary staff, and administrators

SSD schools returned to optional in-person attendance on November 9, 2020, through a phased re-entry model. During the first 6 weeks of study testing (November 23, 2020, to January 19, 2021), the schools were in a hybrid-learning mode, with half of the in-person students present at school Monday–Tuesday and half on Thursday–Friday each week. Students could also opt for full-time virtual learning during this period. Starting January 19, 2021, students could attend school in-person full-time (5 days a week) or opt for full-time virtual learning. At commencement of the study, the lowest in-person student attendance (42%) was in school 3, and the highest in-person attendance (82%) was in school 6 (Table 1). In-person attendance was higher for students in the lower grades.

### Study cohort

A total of 475 participants (416 staff and 59 students) consented to participate in weekly SARS-CoV-2 screening, representing 69% of eligible staff and 13% of eligible students. The median age of all staff participants was 44 (IQR 34–53), 84% were female, and 15% were Black/African-American (Table 2). Staff participation varied from 57 to 83% across the schools. The median age of all student participants was 14 (IQR 11–17), 19% were female, and 24% were Black/African-American (Table 3). Student participation varied from 6 to 22% across the schools.

**Table 2:** Demographics of staff participants in the 6 Special School District of St. Louis County schools

	All schools	School					
		1 (9-12+)	2 (K-8)	3 (9-12+)	4 (K-8)	5 (9-12+)	6 (K-8)
Staff consented	416 (67%)	62 (57%)	87 (83%)	80 (67%)	94 (66%)	43 (61%)	50 (66%)
Median age (IQR)	44 (34–55)	43 (33–51)	42 (33–53)	47 (38–55)	42 (33–49)	48 (36–57)	42 (34–51)
Female (%)	348 (84)	48 (79)	77 (89)	64 (80)	78 (83)	38 (88)	43 (86)
**Race/ethnicity**							
Black/African-American	62 (15)	7 (12)	7 (8)	23 (29)	24 (26)	0 (0)	1 (2)
White Non-Hispanic	325 (78)	49 (80)	75 (86)	54 (68)	60 (64)	41 (95)	46 (92)
Asian	3 (1)	0 (0)	1 (1)	0 (0)	1 (1)	1 (2)	0 (0)
Multiracial	5 (1)	1 (2)	2 (2)	1 (1)	1 (1)	0 (0)	0 (0)
Other	3 (1)	0 (0)	1 (1)	0 (0)	0 (0)	0 (0)	2 (4)
Not provided	17 (4)	4 (7)	1 (1)	2 (3)	8 (9)	1 (2)	1 (2)
**Ethnicity**							
Hispanic/Latino	17 (4)	3 (5)	2 (2)	3 (4)	3 (3)	2 (5)	4 (8)
Unknown	10 (2)	0 (0)	2 (2)	2 (3)	3 (3)	1 (2)	2 (4)
Not provided	23 (6)	5 (8)	8 (9)	2 (3)	5 (5)	2 (5)	1 (2)
**Staff type**							
Teacher/teaching assistant	267 (64)	36 (58)	55 (63)	53 (66)	64 (68)	29 (67)	30 (60)
Administrator/administrative assistant	16 (4)	3 (5)	5 (6)	4 (5)	2 (2)	2 (5)	2 (4)
Nursing staff	20 (5)	4 (7)	5 (6)	3 (4)	2 (2)	5 (12)	1 (2)
Ancillary services	15 (4)	1 (2)	3 (3)	2 (3)	4 (4)	1 (2)	4 (8)
Other	59 (14)	10 (16)	11 (13)	12 (15)	12 (13)	3 (7)	11 (13)
Not provided	37 (9)	8 (13)	8 (9)	6 (8)	10 (11)	3 (7)	2 (4)
**Underlying health conditions**							
None	170 (41)	21 (34)	42 (48)	30 (38)	39 (42)	19 (44)	19 (38)
At least one	206 (50)	32 (53)	36 (41)	46 (58)	42 (45)	22 (51)	28 (56)
Two or more	88 (21)	11 (18)	16 (18)	21 (26)	13 (26)	8 (19)	19 (20)
Vaccination*							
At least one dose	215 (52)	32 (53)	51 (59)	38 (48)	35 (37)	29 (67)	30 (60)
Fully vaccinated	235 (57)	31 (51)	41 (47)	46 (58)	49 (52)	34 (79)	34 (68)

Percentages are indicated in parenthesis

*As of June 1, 2021

**Table 3:** Demographics of student participants in the 6 special school district of St. Louis County schools

	All schools	School					
		1 (9-12+)	2 (K-8)	3 (9-12+)	4 (K-8)	5 (9-12+)	6 (K-8)
Students consented	59 (12)	7 (10)	12 (9)	6 (6)	11 (11)	12 (20)	11 (22)
Median age (IQR)	14 (11–17)	16 (15–19)	12 (11–13)	18 (18–19)	12 (11–13)	17 (15–18)	12 (9–14)
Female (%)	11 (19)	1 (14)	2 (17)	2 (33)	2 (18)	2 (17)	2 (18)
**Student participant race/ethnicity**							
Black/African-American	14 (24)	0 (0)	2 (17)	5 (83)	7 (64)	0 (0)	0 (0)
White non-Hispanic	34 (58)	4 (57)	7 (58)	0 (0)	4 (36)	10 (83)	9 (82)
Asian	1 (2)	0 (0)	1 (8)	0 (0)	0 (0)	0 (0)	0 (0)
Multiracial	6 (10)	1 (14)	2 (17)	1 (17)	0 (0)	1 (8)	1 (9)
Other	1 (2)	0 (0)	0 (0)	0 (0)	0 (0)	0 (0)	1 (9)
Not provided	3 (5)	2 (29)	0 (0)	0 (0)	0 (0)	1 (8)	0 (0)
**Ethnicity**							
Hispanic/Latino	3 (5)	0 (0)	0 (0)	1 (17)	0 (0)	1 (8)	1 (9)
Unknown	1 (2)	1 (14)	0 (0)	0 (0)	0 (0)	0 (0)	0 (0)
Not provided	0 (0)	0 (0)	0 (0)	0 (0)	0 (0)	0 (0)	0 (0)
**Underlying health conditions**							
None	4 (7)	0 (0)	0 (0)	0 (0)	2 (18)	2 (17)	0 (0)
At least one	54 (92)	7 (100)	12 (100)	6 (100)	8 (73)	10 (83)	11 (100)
Multiple	19 (32)	2(29)	3 (25)	4 (67)	3 (27)	5 (42)	2 (18)
Vaccination*							
At least one dose	19 (32)	5 (71)	3 (25)	3 (50)	1 (9)	6 (50)	1 (9)
Fully vaccinated	10 (17)	4 (57)	0 (0)	2 (33)	0 (0)	4 (33)	0 (0)

Percentages are indicated in parenthesis

*As of June 1, 2021

We attempted to test all participants each week. However, adherence to testing among participants decreased over time, from 90 to 63% of consented participants (excluding week 11, during which a snow storm caused school closures) (Supplemental Figure 2). Each week, an average of approximately 46% of all SSD staff and 6% of all SSD students were tested (Supplemental Table 1). Among participants, 12% provided tests for all 24 weeks and 70% provided tests for 12 or more weeks.

**Fig. 1:**
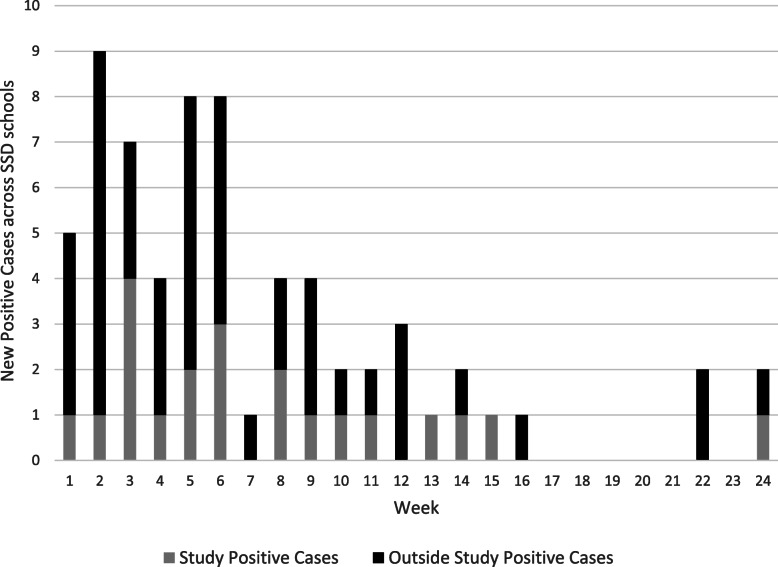
New positive SARS-CoV-2 cases at SSD schools. Shown are number of new positive SARS-CoV-2 cases detected by study weekly screening tests (gray) and new positive SARS-CoV-2 cases detected outside the study through diagnostic testing (black)

During the first 24 weeks of the study, we collected and performed 7289 saliva tests (Supplemental Figure 3). A total of 21 participants (19 staff and 2 students) tested newly positive for SARS-CoV-2 (0.29% of tests performed, excluding repeat positives) (Fig. 1). Following a positive result, the participant’s weekly testing continued but any positives within 12 weeks were not counted as new cases (six participants returned repeat positives; one positive had a recent history of vaccination, with the positive test occurring 10 days after the first dose of vaccine was administered). Across the schools, SSD had a median weekly positivity rate of 0.3% (range 0 to 1.2%). Each SSD school had at least one staff member test positive and in only 2 weeks did more than one school have a positive screening test result. All participating SSD schools remained open, other than the scheduled winter and spring breaks—between weeks 4–5 and 18, respectively, during which no testing took place. During the course of the study, 36 participants withdrew, representing 9% of our participants. Reasons for withdrawal varied, from relocating to a different school not included in the study to becoming vaccinated.

**Fig. 2:**
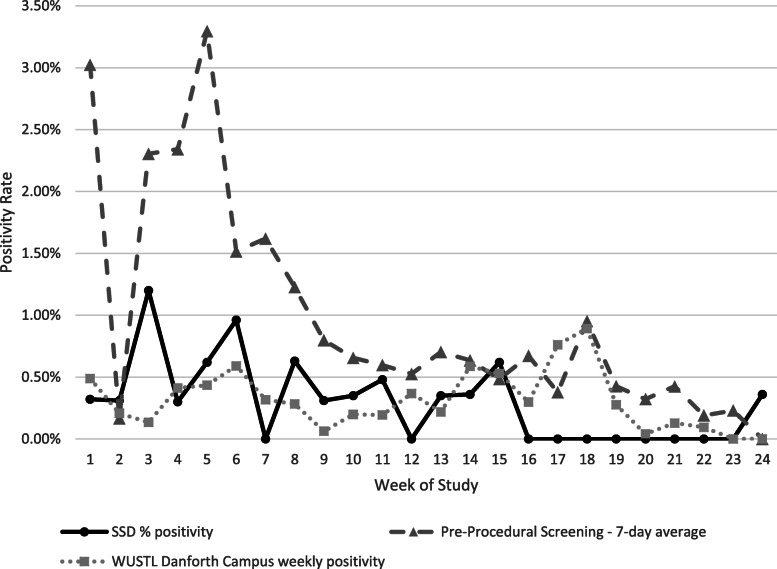
Weekly SARS-CoV-2 screening test positivity rate at SSD schools was not higher than community rates. SSD screening positivity rates (solid black line) compared to (1) asymptomatic pre-procedural test positivity at BJC Hospitals in St. Louis (7-day average) (gray dashed line) and (2) the Washington University in St Louis undergraduate student screening positivity (dotted line) during the same period

To determine whether SSD staff and student positivity was higher than in the community, we compared the participant positivity rate to that of pre-procedure screening tests performed on asymptomatic individuals at BJC Healthcare System in St. Louis during the same period. The SSD participants consistently returned lower weekly positivity rates (0 to 1.2%), compared to screening testing at BJC (0.0 to 3.3%) (Fig. 2) [15]. Furthermore, the SSD positivity rate was comparable to screening of Washington University undergraduate students (Fig. 2). The mean weekly SSD positivity rate was 0.28% of participants compared to mean weekly screening testing positivity rate at the Danforth Campus of 0.31%.

The 19 staff positives represented 4.6% of the 416 staff participants who tested at least once during the study. Outside of the study’s screening tests, an additional 39 SSD staff and 17 students tested positive via symptomatic or post-exposure diagnostic testing. Inclusive of all positive tests, 77 total SARS-CoV-2 positive cases among staff and students were identified.

During the study period, 18.9% (39/206) of staff who did not participate in the study’s screening tests had a positive test, compared with 4.6% (19/416) of staff who participated during at least one study screening test. Meanwhile, of the non-participant students, 2.6% (17/664) had a positive test result during the study period while 3.3% (2/59) of the participating students had a positive during the same period.

### Positive cases

Among the 19 positive staff participants identified during screening testing, 6 resulted from household transmissions, 6 were likely community-based transmissions from attending large gatherings, such as parties, indoor sporting events, and group exercise, and 5 were from other outside-school transmissions (e.g., during vacation). One positive case had both a household exposure (spouse) and an in-school exposure. Only one case was definitively associated with an in-school exposure. During the study period, 103 participants were tested during a quarantine for an in-school exposure and 2 positives (including the participant with both household and school-based exposure) were identified, suggesting a transmission rate of 2% or less. The two participants with possible in-school transmission reported reliably masking and maintaining hand hygiene, but did not wear eye protection. An additional 39 staff members and 17 students tested positive outside of the study, but data were not available to determine if in-school transmission occurred.

Of the 2 positive students in the study, no direct exposures were identified. The first student’s only outside household exposure was attending school. The second student had been to different households, though an epidemiologic link with an ill family member was not identified.

### Mitigation strategies

Aside from the study’s screening tests, masks were mandated and administrators at all 6 schools estimated that 75–100% of staff reliably masked (Table 1). Prior to returning to school, SSD staff reported a widely held assumption that masking would be particularly difficult for children with IDD. However, students were more consistent at wearing masks than expected. For students, masking was initially estimated to be above 50% in all schools. In follow-up discussions near the end of the school year, school staff estimated that the overall rate of mask compliance for students was 70%. The staff reported that masks presented the greatest challenge for students with severe autism, many of whom were rarely able to mask. However, students often responded to continued mask re-enforcement, modeling, and instruction. When students cannot mask consistently, many SSD staff adopted alternatives, including eye protection by students and staff, hand wipes, and adequate room ventilation. Most classrooms allowed students to be spaced at least six feet apart. Ventilation systems were not changed, though schools reported opening windows when possible.

Additional mitigation strategies cited by SSD included the following: implementation of a phased re-entry plan with a gradual transition from hybrid to full-time in-person learning; provision of sufficient personal protective equipment (e.g., masks, gloves, face shields, and hand wipes); reduction of student movement within school; reduction of class sizes to 5–8 students when possible; cleaning between students and maintaining a sanitation schedule for common areas; frequent use of hand wipes for students unable to wash hands; and demonstration of a firm commitment to safety from the highest level of administration, which required canceling or postponing more risky activities (e.g., potlucks, and other indoor gatherings).

Finally, vaccinations became available for staff and students during the study. At each test, participants were asked to provide their vaccination status, dose date(s), and manufacturer. Of all staff participants, 260 (63%) reported receiving at least a first vaccine dose, 235 (56%) a complete vaccine course, and 147 (35%) no vaccination. Students under 16 became eligible to receive the Pfizer vaccine on week 22, and of student participants, 19 (32%) reported receiving at least a first dose.

## Discussion

This study presents the first SARS-CoV-2 screening testing data for students and staff at schools for children with IDD and the use of mitigation strategies to aid the safe reopening of these schools. Throughout the study, which included the COVID-19 pandemic peak, we found no evidence for increased SARS-CoV-2 positivity rates at schools for children with IDD compared to community rates. Furthermore, low rates of transmission were documented among participants with in-school exposures. As with other school studies conducted in schools for typically developing children, most SARS-CoV-2 positive cases were found to result from transmission outside school [8, 9, 16]. In the context of a diagnostic testing regime, attending school in-person has been found to not be associated with an increased likelihood of a positive SARS-CoV-2 test [17].

Our screening testing identified half of the staff SARS-CoV-2 infected cases compared to the number of positive diagnostic tests outside the study, although about two thirds of staff participated in the study. A mandatory screening testing program for Washington University in St. Louis undergraduate students during the same time period also identified nearly half of all known cases [18].

As staff and students were instructed to stay at home when ill, our SSD screening tests should have identified mostly asymptomatic or pre-symptomatic cases. However, when interviewing those who tested positive, many identified COVID-19 symptoms that they misattributed to other causes, such as allergies or sinus infections. Thus, encouraging self-report of mild symptoms with expanded access to convenient, no cost, on-site testing at schools could be an effective strategy to improve early diagnosis and quarantine, and minimize transmission.

As we and others have shown, screening testing may reduce in-school transmission and positivity rate by identifying and isolating cases where infection goes unnoticed or ignored [19]. Additionally, those staff who participated in screening testing were less likely to have COVID-19 during this study period. This result could be due to a number of factors, including study participants are more likely to follow mitigation strategies in and out of school and are less likely to participate in high risk behaviors. However, unless testing is required, voluntary participation is likely to wane over time due to many factors, including decreased sense of urgency as community case rates fall, lack of perceived necessity as individuals become vaccinated, or testing fatigue. More data on screening testing are needed to determine if mandatory school-wide or targeted testing approaches can lower school-based transmission even further in high-risk situations (e.g., caring for unvaccinated and unmasked individuals with IDD).

Although there were major differences in COVID-19 incidence across the St. Louis region at the beginning of the pandemic, at the onset of the study, COVID-19 was widespread with extremely high incidence (50–90 daily cases per 100,000). While positivity rates were similar across all schools, notable differences among in-person school attendance were observed, with lower rates of attendance in schools with a majority of Black/African-American students, where disproportionately higher rates of infection were seen early in the pandemic. Other surveys have demonstrated that Black/African-Americans and Hispanic/Latino parents/guardians were less likely to have their children attend in-person school in Fall 2020 [20]. During the study, a slight increase of the percentage of in-person student attendance occurred, 58 to 65%, across all six schools.

Mitigation strategies implemented at SSD schools alongside our screening testing may explain why in-school transmission was relatively low. SSD school staff attribute successful adoption of masking among students as a significant factor in keeping the positivity rates down and schools open. Beyond masks, adherence to additional mitigation strategies may have limited the in-school impact of COVID-19. However, some mitigation strategies, such as small class sizes and limiting students to small pods, may be less feasible when all students return to school full-time. In addition, it is possible that individuals with greater commitment to safety may choose to return to in-person school as opposed to virtual learning, as others have observed [19], and greater challenges will occur when all children return. School mitigation strategies for children with disabilities, developed in collaboration with SSD staff, are available for dissemination to school administrators, teachers, and parents/guardians at our Safe Return to School for All website [21].

### Limitations

Our study has several limitations. First, participation in screening testing was voluntary and may be biased to include individuals with greater adherence to safety protocols, and therefore, they may have been less likely to test positive than the general school population. Second, we were only able to determine transmission through interviews of positive cases and were not able to verify transmission through mandatory testing or sequencing, which may result in underestimating or misattributing in-school transmission rates. Additionally, we were unable to verify if the cases detected outside of the study were associated with in-school transmission, which may underestimate transmission rates if participants outside the study are systematically different than those who participated. Third, the study took place while in-person student school attendance was reduced both by hybrid scheduling (during the first 6 weeks) and entirely remote learning for 35–42% of children. This could have improved the effectiveness of the mitigation strategies and reduced overall exposures. Finally, most of the study’s testing came before the potentially more transmissible SARS-CoV-2 Delta variant became widespread across the region. While the degree to which the Delta variant may impact transmission is unknown, masking and other currently deployed mitigation strategies will likely remain effective at reducing spread in these school settings.

## Conclusions

During 24 weeks of screening testing staff and students at six SSD of St. Louis County schools coinciding with the winter 2020-21 peak of the COVID-19 pandemic, we found lower rates of SARS-CoV-2 in schools for children with IDD compared to community rates. Screening testing and school adherence to mitigation strategies for managing virus exposure, such as greater than expected masking, may have contributed to the low in-school transmission among participants and lack of increased SARS-CoV-2 positivity compared to community rates. Schools serving students with IDD may consider similar strategies for reducing transmission, including commitments to masking and other mitigation strategies, as well as periodic testing to monitor the effectiveness of COVID-19 mitigation protocols. However, the recent emergence of the more transmissible SARS-CoV-2 Delta variant, which occurred only after the study reporting period, may warrant a cautious interpretation of our data and further study to assess effectiveness of mitigation strategies for this evolving pathogen.

## Supplementary Information

**Additional file 1: Supplemental Figure 1.** Location of the 6 participating Special School District of St. Louis County (SSD) schools. The SSD schools of the study are shown with overlaid regional COVID-19 incidence rates early in the COVID-19 pandemic (March-June 2020) and at study commencement (October-December 2020)(inset). Note: Quintile ranges of new cases per 1,000 estimated residents: Mar-Jun 2020: 0-3.7, 3.7-5.4, 5.4-6.8, 6.8-11, 11-22. Oct-Dec 2020: 0-30, 30-37, 37-41, 41-48, 48-86.

**Additional file 2: Supplemental Figure 2.** SARS-CoV-2 testing participation rates among SSD school staff over 24 weeks of study. Shown are percentages of consented staff and students who were tested for SARS-CoV-2 each week and the percentage of all staff at the 6 participating SSD schools tested each week. Note: a snowstorm during week 11 impacted testing.

**Additional file 3: Supplemental Figure 3.** Total number of SARS-CoV-2 saliva tests performed per week as part of the study. Shown are numbers of weekly screening tests of all participants who were tested for SARS-CoV-2 and the weekly screening tests of students at the 6 participating SSD schools. Note: a snowstorm during week 11 impacted testing.

**Additional file 4: Supplemental Table 1.** Study participation and testing by week.

## Data Availability

Deidentified individual participant data (including data dictionaries) will be made available, in addition to study protocols, the statistical analysis plan, and the informed consent form. The data will be made available upon publication to researchers who provide a methodologically sound proposal for use in achieving the goals of the approved proposal. Proposals should be submitted to gurnettc@wustl.edu.
