# Supplementary material for: SARS-CoV-2 screening testing in schools for children with intellectual and developmental disabilities
Source: J Neurodev Disord. 2021 Sep 1;13:31. doi: 10.1186/s11689-021-09376-z (PMC8407928; doi:10.1186/s11689-021-09376-z)
Supplement: Supplementary file 1 — Additional file 1: Supplemental Figure 1. Location of the 6 participating Special School District of St. Louis County (SSD) schools. The SSD schools of the study are shown with overlaid regional COVID-19 incidence rates early in the COVID-19 pandemic (March-June 2020) and at study commencement (October-December 2020)(inset). Note: Quintile ranges of new cases per 1,000 estimated residents: Mar-Jun 2020: 0-3.7, 3.7-5.4, 5.4-6.8, 6.8-11, 11-22. Oct-Dec 2020: 0-30, 30-37, 37-41, 41-48, 48-86. [file 11689_2021_9376_MOESM1_ESM.docx]

**Supplemental Figure 1. Location of the 6 participating Special School District of St. Louis County (SSD) schools.**


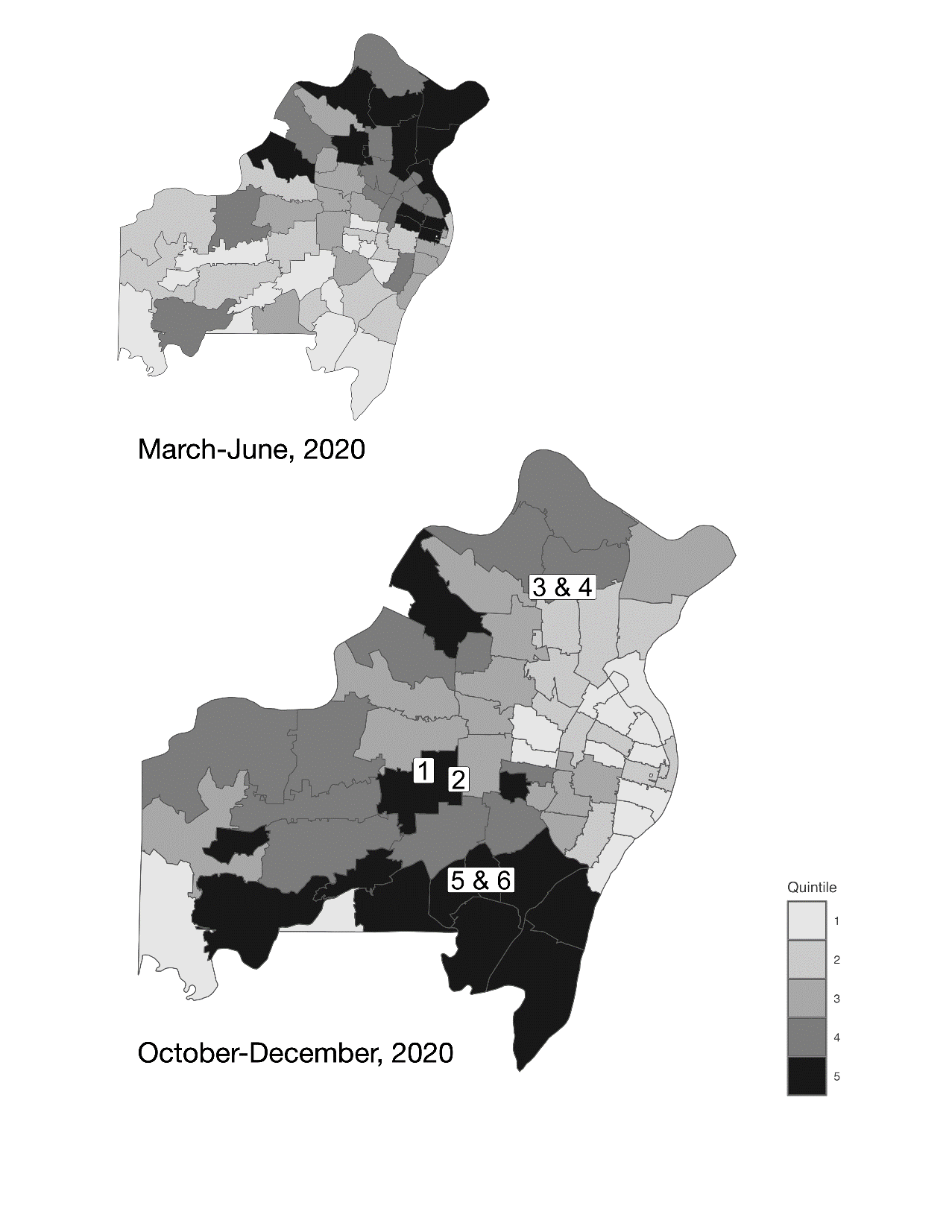


The SSD schools of the study are shown with overlaid regional COVID-19 incidence rates early in the COVID-19 pandemic (March-June 2020) and at study commencement (October-December 2020)(inset).

Note: Quintile ranges of new cases per 1,000 estimated residents:

Mar-Jun 2020: 0-3.7, 3.7-5.4, 5.4-6.8, 6.8-11, 11-22

Oct-Dec 2020: 0-30, 30-37, 37-41, 41-48, 48-86
