# Supplementary material for: SARS-CoV-2 screening testing in schools for children with intellectual and developmental disabilities
Source: J Neurodev Disord. 2021 Sep 1;13:31. doi: 10.1186/s11689-021-09376-z (PMC8407928; doi:10.1186/s11689-021-09376-z)
Supplement: Supplementary file 2 — Additional file 2: Supplemental Figure 2. SARS-CoV-2 testing participation rates among SSD school staff over 24 weeks of study. Shown are percentages of consented staff and students who were tested for SARS-CoV-2 each week and the percentage of all staff at the 6 participating SSD schools tested each week. Note: a snowstorm during week 11 impacted testing. [file 11689_2021_9376_MOESM2_ESM.docx]

**Supplemental Figure 2.** **SARS-CoV-2 testing participation rates among SSD school staff over 24 weeks of study.**

Shown are percentages of consented staff and students who were tested for SARS-CoV-2 each week and the percentage of all staff at the 6 participating SSD schools tested each week. Note: a snowstorm during week 11 impacted testing.
