# Supplementary material for: SARS-CoV-2 screening testing in schools for children with intellectual and developmental disabilities
Source: J Neurodev Disord. 2021 Sep 1;13:31. doi: 10.1186/s11689-021-09376-z (PMC8407928; doi:10.1186/s11689-021-09376-z)
Supplement: Supplementary file 3 — Additional file 3: Supplemental Figure 3. Total number of SARS-CoV-2 saliva tests performed per week as part of the study. Shown are numbers of weekly screening tests of all participants who were tested for SARS-CoV-2 and the weekly screening tests of students at the 6 participating SSD schools. Note: a snowstorm during week 11 impacted testing. [file 11689_2021_9376_MOESM3_ESM.docx]

**Supplemental Figure 3.** **Total number of SARS-CoV-2 saliva tests performed per week as part of the study.**

Shown are numbers of weekly screening tests of all participants who were tested for SARS-CoV-2 and the weekly screening tests of students at the 6 participating SSD schools. Note: a snowstorm during week 11 impacted testing.
