# Supplementary material for: SARS-CoV-2 screening testing in schools for children with intellectual and developmental disabilities
Source: J Neurodev Disord. 2021 Sep 1;13:31. doi: 10.1186/s11689-021-09376-z (PMC8407928; doi:10.1186/s11689-021-09376-z)
Supplement: Supplementary file 4 — Additional file 4: Supplemental Table 1. Study participation and testing by week. [file 11689_2021_9376_MOESM4_ESM.docx]

**Supplemental Table 1: Study participation and testing by week**

| Week | % Staff Consented | % Staff Participants that Tested | Overall % Staff Tested | % Students Participants that Tested | Overall % Students Tested |
| --- | --- | --- | --- | --- | --- |
| 1 | 57% | 90% | 51% | - | 0% |
| 2 | 61% | 87% | 54% | - | 0% |
| 3 | 63% | 87% | 55% | - | 0% |
| 4 | 63% | 85% | 55% | 100% | 0%* |
| 5 | 63% | 84% | 53% | 100% | 0%* |
| 6 | 63% | 82% | 52% | 100% | 0%* |
| 7 | 63% | 81% | 51% | 100% | 0%* |
| 8 | 64% | 81% | 52% | 29% | 0%* |
| 9 | 65% | 81% | 53% | 47% | 2% |
| 10 | 66% | 72% | 47% | 24% | 2% |
| 11 | 66% | 52% | 34% | 50% | 4% |
| 12 | 66% | 73% | 48% | 66% | 6% |
| 13 | 66% | 71% | 47% | 79% | 8% |
| 14 | 66% | 70% | 46% | 82% | 9% |
| 15 | 66% | 69% | 46% | 94% | 11% |
| 16 | 66% | 67% | 44% | 94% | 11% |
| 17 | 64% | 65% | 41% | 87% | 10% |
| 18 | 64% | 64% | 41% | 87% | 10% |
| 19 | 64% | 63% | 40% | 89% | 11% |
| 20 | 64% | 62% | 40% | 78% | 10% |
| 21 | 64% | 61% | 39% | 79% | 10% |
| 22 | 64% | 59% | 38% | 86% | 11% |
| 23 | 64% | 61% | 39% | 86% | 11% |
| 24 | 63% | 60% | 38% | 80% | 10% |

* During weeks 4-8, two students were tested out of 448 students
